# Effect of Polymorphisms in the FCN1, FCN2, and FCN3 Genes on the Susceptibility to Develop Rheumatoid Arthritis: A Systematic Review

**DOI:** 10.1155/2022/1730996

**Published:** 2022-12-15

**Authors:** Sebastián R. Gil-Quiñones, Luz Gutierrez-Castañeda, Lorena Larios-Salazar, Susana Mejia-Mesa, Adriana Motta, David Tovar-Parra

**Affiliations:** ^1^Universidad El Bosque, Bogotá, Colombia; ^2^Research Institute, Basic Science Group, Fundación Universitaria de Ciencias de la Salud, Bogotá, Colombia; ^3^Oncology Department, BioBayter, PBK1422, Malta; ^4^Dermatology Department, Universidad El Bosque, Bogotá, Colombia

## Abstract

Genetic association studies in rheumatoid arthritis conducted in various populations have yielded heterogeneous results. The present systematic review was conducted to synthesize the results of the studies in order to establish the impact of polymorphisms in the ficolin-coding genes FCN1, FCN2, and FCN3 on the susceptibility to develop rheumatoid arthritis. A systematic literature review was performed using the following keywords “gene (FCN1/FCN2/FCN3)”, “Polymorphism/Genetic Variant”, and “rheumatoid arthritis” in different databases until January 2022. Authors assessed articles by title/abstract and then assessed by full text for data extraction. The risk of bias was assessed using the Newcastle-Ottawa scale. Data synthesis was performed qualitatively and quantitatively. A total of 1519 articles were eligible for inclusion in this review, 3 were identified as relevant for the quantitative synthesis with 670 patients and 1019 controls. For the FCN1 gene, an association was found in the dominant and recessive genetic models of the variants rs2989727 (genotype TT = OR: 0.577, 95% CI: 0.430-0.769) and rs1071583 (genotype GG = OR: 1.537, 95% CI: 1.153-2.049, *p* = 0.0032) with the development of rheumatoid arthritis as a protective or susceptibility factor. FCN2 and FCN3 genes did not show association with disease development. The FCN1 gene variants rs2989727 and rs1071583 are associated with the risk of developing rheumatoid arthritis in populations from Brazil and Belgium, but not in FCN2 and FCN3 gene variants.

## 1. Introduction

Rheumatoid arthritis is a chronic, systemic autoimmune disease accompanied by heterogeneous clinical manifestations and persistent inflammation of the synovial membrane leading to damage and destruction of both articular cartilage and adjacent bone [1]. This disease affects between 0.5 and 1.0% of the adult population and is up to 3 times more frequent in women [2]. Serologically, it is characterized by increased acute phase reactants and positivity for rheumatoid factor antibodies (antibodies against immunoglobulin G) and anticitrullinated peptides [3]. Currently, the pathogenesis is not fully elucidated, and both the contributions of environmental factors such as smoking and the involvement of genes in different biological pathways that could confer susceptibility have been identified [3, 4]. In fact, up to 50% of the risk of developing rheumatoid arthritis can be attributed to genetic factors [5].

Both genes associated with innate and adaptive immunity have been identified in rheumatoid arthritis, including major histocompatibility complex class II (HLA: DR, DQ, and DP), such as variants in HLA-DRB1 [3, 4], and some candidate genes such as PTPN22, IL6R, NFKBIE, TNFAIP3, SH2B3, PRKCH, GSDMB, TYK2, and IRAK1, among others, according to genetic association studies such as GWAS (Genome-Wide Association Studies) [6]. Besides, increased levels of ficolins and other complement-associated factors have been found in both synovial fluid and plasma of patients with rheumatoid arthritis and osteoarthritis [7]. Ficolins are a group of proteins involved in the activation of the complement system via the lectin pathway, through interaction with serine proteases associated with mannose-binding lectins in the process of pathogen-associated molecular pattern recognition (PRMs) [8, 9]. Likewise, polymorphisms in the ficolin-1(FCN1) coding gene have been associated with an early onset of type 1 diabetes mellitus in a Brazilian population [10].

Finding new polymorphisms associated to susceptibility to develop rheumatoid arthritis is a significant way to discover new biomarkers with diagnostic and prognostic utility. The present systematic review of the literature focused on synthesizing and evaluating the quality of the available evidence in terms of the risk conferred by the presence of polymorphisms in ficolin-coding genes (FCN1, FCN2, and FCN3).

## 2. Materials and Methods

### 2.1. Selection Criteria

The following inclusion criteria were considered: (a) studies in human population, (b) genetic association studies between rheumatoid arthritis and any polymorphism in the genes coding for ficolins (FCN1, FCN2, and FCN3), and (c) cases diagnosed with rheumatoid arthritis. The studies had to be genetic association studies (case-control studies). No language or year of publication limits were used. Exclusion criteria were duplicated articles, studies with no relevant data, and studies retracted for ethical or scientific accuracy reasons.

### 2.2. Identification of Articles and Search Strategy

A comprehensive search was conducted until December 2021 in the Medline, Web of Science, Scopus, EMBASE, and LILACS databases by combining the search strategies using the PECO (observational studies) question element framework: “Gene (FCN1/FCN2/FCN3)”, “Polymorphism/Genetic Variant”, and “rheumatoid arthritis”. As a search was conducted for each gene and its homonyms, the full search strategy has been stated in full (see table S1). Other potentially useful resources were independently searched in Google Scholar and OpenGrey.

### 2.3. Selection of Studies

A reference software was used to review the articles and eliminated duplicates. Two authors independently screened by title and abstract. Subsequently, the authors reviewed the full texts of the selected articles, in order to meticulously examine compliance with the inclusion and exclusion criteria using a preestablished format. Reasons for exclusion were described for studies not included.

### 2.4. Data Extraction

Data extraction was performed by two authors independently, using a template constructed for this purpose, containing high relevance data for the synthesis, as follows: first author's name, year of publication, number of cases and controls, Hardy-Weinberg equilibrium, clinical diagnosis, sequencing method, gene analyzed, allele and genotypic frequency, clinical significance of the variant analyzed, source of funding, and conflicts of interest. Authors were contacted via email to request missing data.

### 2.5. Risk of Bias and Quality of Evidence Assessment for Individual Studies

The scoring system (0-9) of the Newcastle-Ottawa scale was used to determine the risk of bias for each individual study. Inherent domains of case-control studies such as case/control selection, comparability, and exposure measurement were assessed. In line with the trend in the scientific community for this type of study, a cut-off point of ≥7 was set to be considered low risk of bias. The Newcastle-Ottawa scale was also used to assess the methodological quality of the primary studies.

### 2.6. Statistical Analysis and Data Synthesis

All statistical analyses were performed using Stata16® software. We verified that the studies had calculated the Hardy-Weinberg equilibrium for the control groups of the individual studies; otherwise, this was calculated using a chi-square test, and the relation between the variants in the genes was analyzed by odds ratio (OR) with a confidence interval of 95% (CI 95%). Moreover, analyses of genetic association models (allelic, dominant, recessive, codominant, and overdominant) were performed. The synthesis of the information was performed qualitatively.

## 3. Results and Discussion

### 3.1. Literature Search

**Figure 1:**
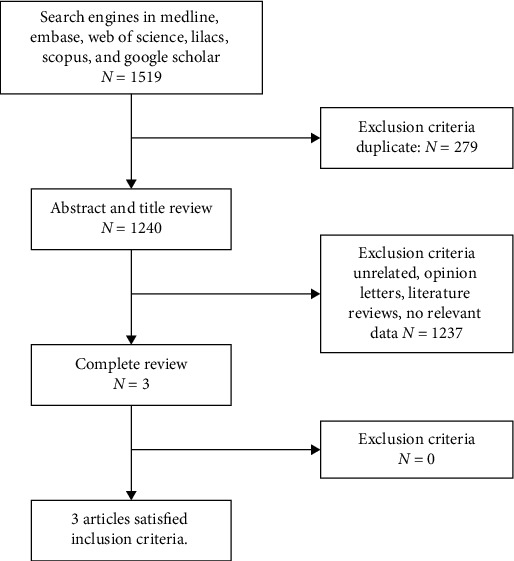
Flow chart of literature screening.

The literature search and screening is illustrated in a PRISMA diagram (Figure 1). Using the combination of terms declared in the search strategy (see list S1) in the various databases Medline, EMBASE, Web of Science, Scopus, Lilacs, and Google Scholar, a total of 1519 articles were obtained. Of these records, 279 duplicates were removed, obtaining 1240 eligible for screening by title and abstract. Of these, 1237 irrelevant records were removed according to the application of selection criteria. Finally, 3 articles were obtained for full-text review, of which all were included in the qualitative and quantitative synthesis.

### 3.2. Characteristics of Included Studies

A total of 3 case-control studies were included, the general data of which are illustrated in Table 1. The distribution of the studies with respect to the gene studied and its variants was as follows: 3/3 for the FCN1 gene, 2/3 for FCN2, and 2/3 for FCN3. These studies were published between 2007 and 2020 and were conducted in the geographical regions of Brazil and Belgium. The origin of the controls was community and hospital-based. Genotyping methods were qPCR (real-time polymerase chain reaction), PCR-SSP (polymerase chain reaction with sequence-specific primers), and reverse hybridization technologies. The score on the Newcastle-Ottawa scale was 9 for all included studies. The specific scores for each risk of bias domain are listed in Table 2. The characteristics of the genetic variants studied and the allele and genotypic frequencies are shown in Table 3.

### 3.3. Risk of Bias Assessment

Applying the Newcastle-Ottawa scale to the included studies found that all scored 9; therefore, they were considered to be of high quality (Table 2). Graphical and statistical assessment of publication bias could not be carried out using forest plot and funnel plot as the number of included studies was less than 10.

### 3.4. Risk-Associated Development of Rheumatoid Arthritis

For the analyses of genetic association models (allelic, dominant, recessive, codominant, and overdominant) in the FCN1, FCN2, and FCN3 genes, we found that the rs2989727 and rs10771583 variants of the FCN1 gene indicated association in allelic, dominant, and recessive models with the development of rheumatoid arthritis as a protective or susceptibility factor: allelic (OR = 0.774, CI 0.671-0.892, *p* = 0.0004), recessive (OR = 0.585, CI 0.452-0.765, *p* = 0.00005), and codominant (OR = 0.577, CI 0.430-0.769, *p* = 0.0002) and allelic (OR = 1.316, CI 1.123-1.546, *p* = 0.0008), recessive (OR = 1.346, CI 1.062-1.706, *p* = 0.013), and codominant (OR = 1.719, CI 1.246-2.372, and *p* = 0.0009), respectively (see Table 4).

On the other hand, the study by Pieczarka et al. found an association of rs10120023 with the risk of developing rheumatoid arthritis in a group of 148 cases and 160 controls based on an allelic model which confers an increased risk to the G allele (OR: 1.47; 95% CI: 1.02-2.12; *p* value: 0.041); conversely, the A allele confers a protective effect (OR: 0.59; 95% CI: 0.37-0.95; *p* value: 0.041).

Other polymorphisms analyzed but not associated were rs2989727, rs17039495, rs10117466, and rs10858293 [11]. In contrast, Addobbati et al. studied 2 polymorphisms in the FCN1 gene (rs2989727 and rs1071583) in the analysis of 137 cases and 264 controls, without finding a statistically significant association between their presence and the risk of developing rheumatoid arthritis [12].

Finally, Vander Cruyssen et al. found statistically significant associations from 338 cases and 595 controls for the rs2989727 and rs10771583 variants. The former was a codominant model where the GG genotype conferred an increased risk (OR: 2.24; 95% CI: 1.45-3.46), and the latter variant was studied using the same model, the risk genotype being AA (OR: 1.94; 95% CI: 1.25-3.00) [13].

In contrast, for the association analyses implementing the genetic models in the FCN2 gene variants (rs7851696, rs17514136, and rs3124954, among others) and the FCN3 gene variants (rs532781899, rs28362807, rs4494157, and rs3813800), no association was found between the variants analyzed in the study and the risk of developing rheumatoid arthritis.

## 4. Discussion

Rheumatoid arthritis is a chronic multifactorial autoimmune disease characterized by the presence of persistent inflammation on the synovial membrane, which has an important genetic component and a heritability of approximately 65% [14]. The genes most implicated have been those encoding major histocompatibility complex class II proteins (HLA DR, DQ, and DP) in up to 60% [15, 16], with the HLA-DRB1 alleles accounting for a large part of the risk of the disease [3, 4, 6]. Another important factors influencing susceptibility to develop rheumatoid arthritis, apart from adaptive immunity, are proteins related to innate immunity such as those associated with the complement pathway [7, 17]. Ficolins are involved in the lectin pathways, and their serum concentration has been associated with markers of disease activity such as DAS-28 (Disease Activity Score in 28 Joints) [17].

As a result, the role of genes coding for the initiator proteins of the complement lectin pathway began to be studied, of which the following have been identified: FCN1, FCN2, FCN3, and MBL2 [18], whose protein products serve as recognition molecules against pathogens, thus enabling their opsonisation [8]. Within studies of the FCN1, FCN2, and FCN3 genes, Pieczarka et al. found an association between rheumatoid arthritis and the rs10120023 variant of the FCN1 gene (allele G = OR: 1.47; 95% CI: 1.02-2.12; *p* value: 0.041) [11], and Vander Cruyssen et al. found statistically significant associations with the rs2989727 variants (genotype GG = OR: 2.24; 95% CI: 1.45-2.12; *p* value: 0.041) and rs1071583 (genotype AA = OR: 1.94; 95% CI: 1.25-3.00) [13], as the protein encoded by this gene is mainly present in neutrophils and bone marrow [18, 19]. Variants in this gene have been associated with diseases such as systemic lupus erythematosus, leprosy, and diabetes [10, 12, 20, 21].

In the analyses of the studies in population such as Belgium and Brazil, FNC1 gene variants were associated with rheumatoid arthritis (OR = 0.774, 95%CI = 0.671 − 0.892, and *p* = 0.0004 and OR = 1.537, 95%CI = 1.153 − 2.049, and *p* = 0.0032) as a protective or risk factor in the genetic models analyzed. In contrast, no association was found between the FCN2 and FCN3 genes with the development of rheumatoid arthritis, even though variants in the FNC2 promoter have been associated with the presence of lupus nephritis in pediatric patients with systemic lupus erythematosus, such is the case of the rs3124952 variant (genotype GG = OR: 2.6; 95% CI: 1.4-4.78; *p* value = 0.006) and rs17514136 (AA genotype = OR: 3.12; 95% CI: 1.25-7.84; *p* value = 0.024).

In addition, the rs7851696 variant in the FCN2 gene has been associated with delayed graft function and acute graft rejection in renal transplant patients (T allele = OR 1.71; 95% CI: 1.02-2.87, *p* value = 0.048) [22]; in other diseases such as vasculitis, ficolin-M expression is elevated in peripheral blood mononuclear cells and inflamed areas [23] and severe pneumonia in the Egyptian population [24].

For the rs4494157 variant, it has been associated with an increased susceptibility to rheumatic fever with or without rheumatic heart disease (allele A = OR: 2.93, *p* value: 0.0002 and OR = 2.23, *p* value = 0.008, respectively) [25], and the +1637delC variant, involving a heterozygous deletion state in the gene and consequent ficolin-3 deficiency, was associated with an increased risk of developing the disease (OR: 8.0; 95% CI 1.01-63.0; *p* value < 0.05%) [26].

## 5. Conclusions

Both polymorphisms in the FCN1 gene (rs2989727 and rs1071583), but not in FCN2 and FCN3, are associated with the risk of developing rheumatoid arthritis in populations from Brazil and Belgium. The association between these variants and susceptibility to rheumatoid arthritis needs to be further investigated using more robust research designs in order to clarify a more precise causal association. Further studies are needed to complement the findings obtained in a quantitative synthesis from a meta-analysis.

## Figures and Tables

**Table 1:** Data from studies included in the systematic review.

Author	Year	Gene	Genetic variants	Geographic region	Study type	Source of controls	NOS^∗^	Number of cases	Number of controls
Pieczarka et al. [11]	2015	FCN1 FCN2	rs2989727 rs1071583 rs17514136 rs3124954 rs7851696	Brazil	Cases and controls	Community	9	184	264

Addobbati et al. [12]	2020	FCN1 FCN3	rs2989727 rs10120023 rs17039495 rs10117466 rs10858293 rs532781899 rs28362807 rs4494157	Brazil	Cases and controls	Community	9	148	160

Vander Cruyssen et al. [13]	2007	FCN1 FCN2 FCN3	rs2989727 rs1071583 rs7865453 ss32469537 ss32469544 rs7851696 rs3813800	Belgium	Cases and controls	Community	9	338	595

The variants are described according to their RS code (RefSNP) assigned in dbSNP (genetic variant database of the US National Institutes of Health). Available at https://www.ncbi.nlm.nih.gov/snp/. ^∗^Score on the Newcastle-Ottawa scale (NOS).

**Table 2:** Risk of bias assessment of included studies based on the Newcastle-Ottawa scale.

Study		Newcastle-Ottawa scale domains								
		Selection				Comparability	Exposition			Total
First author	Year	Proper case definition	Representivity of the cases	Selection of controls	Definition of controls	Control of important factors	Exposure assessment	Same method for the measurement of all subjects	Nonresponse rate	
Addobbati C	2015	1	1	1	1	2	1	1	1	9
Pieczarka C	2020	1	1	1	1	2	1	1	1	9
Vander Cruyssen B	2007	1	1	1	1	2	1	1	1	9

A score greater than or equal to 7 indicates low risk of bias and good methodological quality of the case-control study in the domains of selection, comparability, and exposure.

**Table 3:** Allelic and genotypic frequencies of cases and controls.

Study data						Cases							Controls						
Author	Year	Gene	RS	Alleles	Risk allele	Cases	Total alleles	Allele ref^∗^	Risk allele	WT	HT	HH	Control	Total alleles	Allele ref^∗^	Risk allele	WT	HT	HH
Addobbati C	2015	FCN1	rs2989727	C/T	T	137	274	124	150	27	70	40	264	528	206	322	40	126	98
			rs1071583	C/T	T	137	274	165	109	50	65	22	264	528	341	187	109	123	132
		FCN2	rs17514136	A/G	G	184	368	286	82	108	70	6	264	528	401	127	156	89	19
			rs3124954	C/T	T	184	368	241	127	84	73	27	264	528	345	183	116	113	35
			rs7851696	G/T	T	184	368	318	50	136	46	2	264	528	458	70	197	64	3

Pieczarka C	2020	FCN1	rs2989727	G/A	A	148	296	152	144	35	82	31	159	318	147	171	38	71	50
			rs10120023	G/A	A	148	296	230	66	89	52	7	159	318	224	94	74	76	9
			rs17039495	G/A	A	148	296	288	8	140	8	0	159	318	316	2	157	2	0
			rs10117466	C/A	A	148	296	234	62	93	48	7	159	318	232	86	81	70	8
			rs10858293	G/T	T	148	296	226	70	83	60	5	159	318	226	92	78	70	11
		FCN3	rs532781899	g.1637C/g.1637del	g.1637del	148	296	290	6	142	6	—	160	320	312	8	153	7	—
			rs28362807	g.3524_3532ins	g.3524_3532ins	148	296	210	86	75	60	13	160	320	244	76	96	52	12
			rs4494157	g.4473C/g.4473A	g.4473A	148	296	215	81	79	57	12	160	320	250	70	102	46	12

Vander Cruyssen B	2007	FCN1	rs2989727	G/A	A	338	676	226	450	145	162	31	595	1190	476	714	220	268	107
			rs1071583	A/G	G	338	676	216	460	152	156	30	595	1190	464	726	226	274	95
		FCN2	rs7865453	A/C	C	338	676	616	60	—	—	—	595	1190	1048	142	—	—	—
			rs32469537	A/G	G	338	676	480	196	—	—	—	595	1190	880	310	—	—	—
			rs17549193	C/T	T	338	676	466	210	—	—	—	595	1190	880	310	—	—	—
			rs7851696	G/T	T	338	676	608	68	—	—	—	595	1190	1036	154	—	—	—
		FCN3	rs3813800	C/G	G	338	676	676	0	—	—	—	595	1190	1178	12	—	—	—

WT: wild-type genotype or homozygous for the major allele; HT: heterozygous genotype; HH: genotype homozygous for the risk allele.

**Table 4:** Genotype and allele distributions of variants FCN1 gene in cases and control groups.

FCN1 (rs2989727)							
Model	Genotype allele	Cases *n* = 623	Control *n* = 1019	OR	95% CI	*p* value	Chi^2^
Codominant	C/C	207	298	1	Reference		
	C/T	314	465	0.965	0.768-1.213	0.765	0.088
	T/T	102	255	0.577	0.430-0.769	0.0002	14.025
Dominant	C/C	207	298	0.831	0.671-1.030	0.0922	2.835
	C/T+T/T	416	720				
Recessive	C/C+C/T	521	763	0.585	0.452-0.756	0.00005	17.093
	T/T	102	255				
Overdominant	C/C+T/T	309	553	1.208	0.989-1.475	0.0629	3.457
	C/T	314	465				
Allele	T	518	975	0.774	0.671-0.892	0.0004	12.431

FCN1 (rs1071583)							
Model	Genotype allele	Cases *n* = 475	Control *n* = 859	OR	95% CI	*p* value	Chi^2^
Codominant	T/T	80	204	1	Reference		
	T/C	221	397	1.419	1.044-1.928	0.024	5.043
	C/C	174	258	1.719	1.246-2.372	0.0009	10.975
Dominant	T/T	80	204	1.537	1.153-2.049	0.0032	8.706
	T/C+C/C	395	655				
Recessive	T/T+T/C	301	601	1.346	1.062-1.706	0.013	6.078
	C/C	174	258				
Overdominant	T/T+C/C	254	462	1.012	0.808-1.267	0.913	0.011
	T/C	221	397				
Allele	C	569	913	1.316	1.123-1.546	0.0008	11.292

## Data Availability

The extracted data used to support the findings of this study are mainly included within the article and supplementary data.

## Supplementary Materials

Supplementary Materials See Table S1 literature search strategies in different databases and List S1 for combination terms declared in the search strategy.
