# Supplementary material for: Effect of Polymorphisms in the FCN1, FCN2, and FCN3 Genes on the Susceptibility to Develop Rheumatoid Arthritis: A Systematic Review
Source: Int J Rheumatol. 2022 Dec 15;2022:1730996. doi: 10.1155/2022/1730996 (PMC9780007; doi:10.1155/2022/1730996)
Supplement: Supplementary Materials — See Table S1 literature search strategies in different databases and List S1 for combination terms declared in the search strategy. [file 1730996.f1.docx]

**SUPPLEMENTARY MATERIAL**

Table 1: Literature Search Strategies.

| **PICO ELEMENT** | **DESCRIPTION PICO ELEMENT** | **TERMS (MESH/DECS)** | **NATURAL TERMS** |
| --- | --- | --- | --- |
| **Population** | **General population, unspecified because they are genetic studies.** | **Not applicable** | **Not applicable** |
| **Intervention** | **Presence of polymorphisms in the ficolin coding genes (FCN1, FCN2 and FCN3).** | **Polymorphism, Genetics**  **Genetic Polymorphism**  **Ficolins**  **Ficolin** | **Genetic Polymorphism Genetic Polymorphisms Gene Polymorphism Polymorphisms Polimorfismo FCN1  FCN2 FCN3** |
| **Comparador** | **Not applicable** | **Not applicable** | **Not applicable** |
| **Outcome** | **Risk/susceptibility to develop rheumatoid arthritis** | **Arthritis, Rheumatoid Artritis Reumatoide** | **Rheumatoid arthritis Artritis reumatoidea** |

**List S1. Combination of terms declared in the search strategy**

1. **SUPPLEMENT 1.1: MEDLINE DATABASE (VIA PUBMED)**

[1]. (((arthritis, rheumatoid [MeSH Terms]) OR rheumatoid arthritis) AND ((polymorphism, genetic [MeSH Terms]) OR polymorphism) AND (ficolin OR FCN*))

[2]. (((arthritis, rheumatoid [MeSH Terms]) OR rheumatoid arthritis) AND ((polymorphism, genetic [MeSH Terms]) OR polymorphism)

1. **SUPPLEMENT 1.2: LILACS DATABASE (VIA BVS)**

[1]. (artritis reumatoide) AND (polimorfismo genético OR polimorfismo) AND (ficolina OR FCN)

[2]. (artritis reumatoide) AND (polimorfismo genético OR polimorfismo)

1. **SUPLEMENTO 1.3: WEB DE LA CIENCIA (VÍA PORTAL WoS)**

[1]. ((ALL=(rheumatoid arthritis )) AND ALL=(polymorphism OR polymorphisms OR genetic polymorphism)) AND ALL=(ficolin OR FCN)

1. **SUPPLEMENT 1.4: SCOPUS (VIA ELSEVIER.COM)**

[1]. ( ( rheumatoid AND arthritis ) AND ( polymorphism* ) AND ( ficolin OR fcn ) )

1. **SUPPLEMENT 1.5: EMBASE (VIA EMBASE.COM)**

[1]. ('rheumatoid arthritis'/exp OR 'rheumatoid arthritis') AND 'genetic polymorphism' AND (ficolin OR 'fcn1 gene' OR 'fcn2 gene' OR 'fcn3 gene')

[2]. ('rheumatoid arthritis'/exp OR 'rheumatoid arthritis') AND 'genetic polymorphism' AND ('ficolin')

1. **SUPPLEMENT 1.6: GOOGLE SCHOLAR (VIA GOOGLE.COM)**

[1]. rheumatoid arthritis ficolin "polymorphism"
